# Cases by Dr. Wm. Harris

**Published:** 1856-02

**Authors:** 


### THE
### MEDICAL EXAMINER.

NEW SERIES.—NO. CXXXIV.—FEBRUARY, 1856.

### ORIGINAL COMMUNICATIONS.

### CASES BY DR. WM. HARRIS.

*Induction of Labor at the Seventh Month for the relief of Obstinate Vomiting.*

In the month of July, 1855, Mrs. ——, a lady of about thirty-four years of age, pregnant with her fourth child, was at Cape May, and while there, in walking from the bath to the house, was tripped up by the long skirt of her bathing dress, and thrown violently to the ground. In falling she struck her abdomen with force, which casualty was followed by very serious consequences. She never had a well day after the fall, and thus the way was prepared for the case, an account of which is now presented.

For three months after this occurrence, Mrs. —— suffered intensely from neuralgia of the womb, and was confined to her bed during that time, except when she left it to make trial of her strength and power of endurance. In every such instance her sufferings were so severe that she was obliged to return to bed. During the last fortnight of this period, her disrelish for every kind of food was so great that she could retain nothing on her stomach for a moment. The ordinary remedies were resorted to in vain. Every effort which could be suggested for her relief was made, but she still remained in the same state of prostration and suffering. At this crisis I was led confidently to the opinion that in order to save her life it would be necessary to hasten her labor, and accordingly proposed this course to the family, when it was thought best to call in a consulting physician.

Prof. Joseph Carson was selected and called in. He stated that a case precisely similar had fallen under his observation which was relieved by administering a wine glass full of Liebig's soup, per anum, every two hours, and recommended that the same treatment should be used in the present case. This was accordingly done, but without the least success.

After a lapse of twenty-four hours, during which these means were tried, Dr. Carson called again and found the patient so completely prostrated that he was forced to the conclusion that nothing but a premature delivery could save her life.

It was now the beginning of the seventh month of utero-gestation, and on the 23d of October, 1855, I made a per vaginam examination with Dr. Carson, in the course of which I distinguished a hand, a foot, and the head of a child. From the fact that I felt distinctly a foot before I came to the head, I at first supposed that there were twins, but this proved afterwards not to be the case.

At 8 o'clock in the evening I began to dilate the os uteri with my index finger, by pressing it forcibly in every direction. It was dilated two inches in about two hours, when I plunged a silver catheter through the membranes and let out a very large quantity of water, perhaps half a gallon. This afforded instant relief to the patient. She no longer complained of the deadly nausea, and took a wine glass full of wine whey, which she retained without inconvenience. In the progress of the labor I succeeded in putting the hand back, and afterwards the foot, and now the head fairly engaged in the superior strait. Labor pains fully set in, the progress of the child was regular, and about two o'clock in the morning it was born, and lived about three hours. The patient never suffered with nausea or vomiting after the membranes were ruptured and the waters evacuated. After she was put to bed she ate the breast of a partridge, rice, cream toast, and drank a cup of tea, all of which she retained on her stomach, with great improvement of comfortable feeling. In the evening, however, she had a return of neuralgia of the womb, but accompanied by neither nausea or vomiting. She was relieved by taking a mixture of morphia and camphor water, one grain to the ounce. Of this she took a dessert spoonful every hour. She also took a wine glass full of wine whey hourly, and was relieved and slept well during the latter part of the night. The next day she was very well, ate heartily of light nutritous food, and continued in good spirits until evening, when she had another attack of neuralgia, lighter than that of the previous evening, which was relieved by the use of the same remedies. For several evenings she had a return, but at each time with less severity, until they entirely disappeared. She continued to improve, so that at the end of three weeks she left her bed, and at the end of six weeks she went to New York on an excursion of pleasure.

Could the fall described in the introduction to this article have knocked the child into the position in which I found it: and could this position of the child have caused the violent neuralgia and deadly nausea and vomiting which the patient so long experieneed ? Or did it arise from the distension of the womb by the inordinate quantity of liquor amnii which was present ?

### *A case of Imperforate Anus—Operation—Cure.*

Mrs ——, the wife of a merchant, was delivered on the 3d of December last, of a fine healthy son, she and her son doing apparently very well. On the morning of the 4th, I was informed that the infant's bowels had not been opened, whereupon, I ordered a teaspoonful of castor oil, and if it did not operate in twelve hours, another.

About 10 o'clock in the evening of the second day after its birth, the father of the child called at my house to inform me that the medicine had not operated, and that he occasionally vomited. I directed an enema, consisting of a gill of warm water and a dessert spoonful of castor oil. On the following morning, I was told that the enema did not go into the child's bowel, but returned along the side of the nozzle of the syringe, and that the child still continued to be sick at the stomach, and occasionally vomited.

Immediately suspecting an imperforate anus, I introduced my little finger into the rectum, and found it as I supposed, completely obstructed about half an inch up. Professor Neill was immediately sent for, who promptly came to my assistance.* As

* In the course of the consultation, we remarked that nearly all such cases terminated fatally. A few years ago, I had one, the result of which was most the result of our consultation, we determined to proceed very cautiously; accordingly, Doctor Neill introduced his little finger and dilated the part as much as he could, after which he passed a small sized trochar very slowly through the obstruction into the gut above. On withdrawing the stilet, there flowed after it nearly a gill of meconium. He then stretched the opening thus made by introducing two directors, and by pressing them in different directions he dilated the wound without producing any hæmorrhage. The operation was thus far successful, and the contents of the bowels, being in a fluid state, were easily evacuated several times a day. We called daily for several days, and finding there was a disposition in the part to close up, Dr. Neill again introduced the probes or directors, and dilated the aperture. The child being tolerably comfortable, Dr. Neil discontinued his visits.

About a week after this, I found that the fæces were passed with great difficulty, nor could I with ease introduce a small probe, and, in attempting to introduce the smallest sized gum elastic male catheter, such as are used for infants, I entirely failed. I determined, however, not to operate again, as a distinguished surgeon, a few days since, had a similar case in which a second operation proved fatal; the child having bled to death.

Having made up my mind to attempt to dilate it with metallic bougies, I carried with me six silver ones, which I had used before in dilating strictures of the urethra. They were of different sizes, the two ends of each of them being also of different widths. I began with the smallest end of the smallest sized one, and introduced it with difficulty; then the next day the larger end. Each introduction was followed by a few drops of blood; I thus proceeded cautiously until I succeeded in passing the larger end of the largest size bougie without any difficulty or loss of blood. I intend to continue this operation once a week for a few weeks, though I consider the child perfectly cured.

unfortunate. I called in Professor Horner, who plunged a *large trochar* through the obstruction into the cavity of the abdomen, as was ascertained by introducing a gum elastic catheter. The child passed a few days of pitiable suffering and then died. The parents positively refused a *post-mortem* examination, by which we could have ascertained whether the trochar passed through the side of the gut, or whether there was a mal-formation, and the intestine not continuous.

